# Pancreatic Cancer Meets Human Microbiota: Close Encounters of the Third Kind

**DOI:** 10.3390/cancers13061231

**Published:** 2021-03-11

**Authors:** Tatjana Arsenijevic, Remy Nicolle, Christelle Bouchart, Nicky D’Haene, Pieter Demetter, Francesco Puleo, Jean-Luc Van Laethem

**Affiliations:** 1 Laboratory of Experimental Gastroenterology, Université Libre de Bruxelles, Route de Lennik 808, 1070 Brussels, Belgium; tatjana.arsenijevic@erasme.ulb.ac.be (T.A.); francesco_puleo@hotmail.com (F.P.); 2 Department of Gastroenterology, Hepatology and Digestive Oncology, Hôpital Erasme, Université Libre de Bruxelles, Route de Lennik 808, 1070 Brussels, Belgium; 3 Programme Cartes d’Identité des Tumeurs (CIT), Ligue Nationale Contre le Cancer, 75013 Paris, France; remy.nicolle@ligue-cancer.net; 4 Department of Radiation-Oncology, Institut Jules Bordet, Université Libre de Bruxelles, 1000 Brussels, Belgium; christelle.bouchart@bordet.be; 5 Department of Pathology, Erasme Hospital, Université Libre de Bruxelles (ULB), 1070 Brussels, Belgium; Nicky.d.Haene@erasme.ulb.ac.be; 6 Department of Pathology, Institut Jules Bordet, Université Libre de Bruxelles, 1000 Brussels, Belgium; pieter.demetter@bordet.be; 7 Delta Hospital, Centre Hopitalier Interrégional Edith Cavell (CHIREC), 1160 Brussels, Belgium

**Keywords:** pancreatic ductal adenocarcinoma, microbiota, prognostic and predictive biomarkers, resistance to therapy

## Abstract

**Simple Summary:**

The microorganisms colonizing the epithelial surfaces of the human body, called microbiota, have been shown to influence the initiation, progression and response to therapy of many solid tumors, including pancreatic ductal adenocarcinoma, the most prominent form of pancreatic cancer. Here, we summarize the current knowledge about the influence of oral, gut and intratumoral microbiota on pancreatic ductal adenocarcinoma development and chemoresistance.

**Abstract:**

Pancreatic ductal adenocarcinoma (PDAC) remains one of the most lethal types of cancer with a dismal prognosis. The five-year survival rate has not changed significantly in over 40 years. Current first-line treatments only offer a modest increase in overall survival in unselected populations, and there is an urgent need to personalize treatment in this aggressive disease and develop new therapeutic strategies. Evolving evidence suggests that the human microbiome impacts cancerogenesis and cancer resistance to therapy. The mechanism of action and interaction of microbiome and PDAC is still under investigation. Direct and indirect effects have been proposed, and the use of several microbiome signatures as predictive and prognostic biomarkers for pancreatic cancer are opening new therapeutic horizons. In this review, we provide an overview for the clinicians of studies describing the influence and associations of oral, gastrointestinal and intratumoral microbiota on PDAC development, progression and resistance to therapy and the potential use of microbiota as a diagnostic, prognostic and predictive biomarker for PDAC.

## 1. Introduction

Pancreatic ductal adenocarcinoma (PDAC), the most prevalent type of pancreatic cancer, counts among the deadliest cancers. Only 9% of all patients diagnosed with PDAC are expected to survive five years post-diagnosis [1]. Surgery is the only path to cure PDAC, but only a small number of patients present with a resectable tumor at the time of diagnosis. Moreover, early detection is hampered by the vagueness of symptoms and the lack of specific clinical markers of the early stages of the disease. The diagnosis heavily relies on endoscopic ultrasound (EUS)/fine needle aspiration (FNA) biopsies combined with advanced pancreatic imaging techniques. There is an urgent need to develop additional, less-invasive diagnostic methods to be able to detect PDAC at early, resectable stages.

Although the complete etiology of PDAC has not been fully understood, several factors are known for increasing the overall risk. They include smoking, obesity, dietary habits, type II diabetes mellitus and alcohol consumption [2]. It is estimated that 10% to 20% of all PDAC can be attributed to smoking [3,4,5]. Obesity and alcohol consumption have also been positively correlated with PDAC [2,6,7]. Type II diabetes mellitus is likely the third risk factor for PDAC after cigarette smoking and obesity. Individuals with elevated blood glucose and/or diagnosed long-term type II diabetes mellitus present for more than 10 years have an increased risk of PDAC [8,9,10] while recent onset diabetes mellitus might be an initial indicator of PDAC [11]. Besides those environmental, modifiable risk factors, some non-modifiable factors have been identified like the age (pancreatic cancer risk increases with age), gender (men have been shown to be slightly more susceptible to develop pancreatic cancer than women) and non-O blood types (slightly increased risk of PDAC is detected in individuals with a non-O blood group) [2,12,13,14]. In addition, it is estimated that up to 10% of all PDAC cases are attributed to a familial syndrome, and this number is predicted to increase as new, complex combinations of factors are discovered [15]. Besides the above-mentioned environmental and genetic risk factors, an increasing number of recent reports are showing an association between the composition of the human microbiota and PDAC. Microbiota are communities of commensal, symbiotic and pathogenic microorganisms, including bacteria, archaea, protists, fungi and viruses that inhabit the human body. Human microbiota consists of 10–100 trillion microorganisms that are harbored by each person [16,17]. Microbiota can be found in the skin, oral and nasal cavity and urogenital tract; however, the bacterial count in intestinal organs (gut microbiota) outnumbers by far other body parts [18]. In contrast to the human genome which consists of approximately 20,000 inherited genes, the human microbiome (the genetic material of all the microbes in one person’s microbiota) is acquired, contains about 10 to 20 million genes and changes throughout life influenced by various environmental and pharmacological factors [19,20,21]. Microbial diversity is assessed using two types of measures: alpha diversity, defined as the number of unique taxa and their distribution in a specimen, and beta diversity, which is assessing the difference in species composition of two groups of specimens.

An imbalance in the microbial composition and functionality, a state called dysbiosis, and mucosal barrier impairment are associated with diverse diseases, including pancreatic diseases such as acute and chronic pancreatitis and PDAC [22]. Dysbiosis is often associated with reduced microbiome diversity [23].

**Figure 1:**
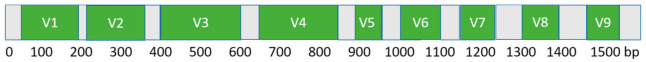
Schematic representation of the bacterial 16S rRNA gene.

The most widespread technique used to identify the bacterial composition in a specimen is targeted 16S rRNA amplicon sequencing. The 16S rRNA gene is composed of conserved, unchanged domains, present in all bacterial species and nine different hypervariable regions V1 to V9 (Figure 1). Hypervariable regions are a blueprint for every bacteria and are widely used in the identification, classification and phylogenetic analysis of various bacteria. To detect specific species, sets of primers are designed based on the sequence information of the variable regions. The accuracy of this method strongly depends on the choice of primer pairs and, in particular, on the balance between efficiency, specificity and sensitivity in the amplification of the different V1–V9 bacterial 16S sequences contained in a sample [24].

**Figure 2:**
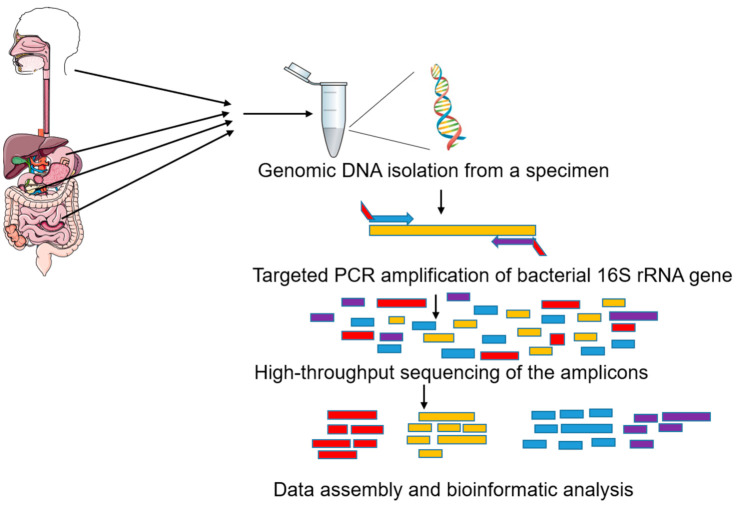
Workflow for identification of microbiota in a specimen by targeted 16S rRNA amplicon sequencing.

After a specific region of the 16S rRNA gene is amplified from the extracted genomic DNA from a specimen and sequenced, data are compared with reference databases to determine the genus and, to some extent, species of bacteria present in the specimen (Figure 2). For the detection of mycobiota (collection of fungi in a specimen), the workflow is identical, except the 18S rRNA gene and their internal transcribed spacer (ITS) sequence variants are used for detection. As it is frequently difficult to distinguish species using rRNA sequences, the quantification is associated with an operational taxonomic unit (OTU), corresponding to the most specific taxonomic level regrouping all sub-species with an indistinguishable sequence.

In this review, we provide an overview of data obtained from human and animal studies describing the influence and associations of oral, gastrointestinal and intratumoral microbiota on PDAC development, progression and resistance to therapy and the potential use of microbiota as a diagnostic, prognostic and predictive biomarker.

## 2. Oral Microbiota and PDAC Risk

The oral cavity is an extensive reservoir of bacteria composed of more than 700 species [25]. Periodontitis is an inflammatory disease of the oral cavity, and there are several microbes described to be strongly implicated in periodontitis development, including *Porphyromonas gingivalis* and *Aggregatibacter actinomycetemcomitans* [26]. Several epidemiological studies have reported the relationship between periodontitis and tooth loss and an increased risk of PDAC [27,28,29,30,31]. A large prospective study has shown that periodontal disease was associated with a 64% higher risk for PDAC. The relative risk was even twice as high in non-smokers, ruling out the possibility that this association was confounded with smoking [28].

The unexpected observed link between periodontal disease and PDAC initiated several observational studies on the impact of the composition and changes in the oral microbiota composition in PDAC etiology (Table 1).

In 2012, Farrell et al. compared microbiota composition in saliva in a discovery group of 10 PDAC patients and 10 matched healthy controls and validated their findings in an independent cohort of 28 PDAC patients, 28 healthy controls and 27 patients with chronic pancreatitis (CP). 16S rRNA amplicons were hybridized on Human Oral Microbe Identification Microarray (HOMIM) and further validated by qPCR. Two bacteria, *Neisseria elongata and Streptococcus mitis*, were found to be lower and *Granulicatella* *adiacens* elevated in PDAC cases compared to healthy controls in both datasets, proposing the utility of the abundance of these bacterias as a specific biomarker for PDAC [32]. The main limitation of this study is a small sample size that did not allow for a subgroup analysis to assess whether the associations are consistent across different populations defined by factors such as race, ethnicity and smoking status.

Another study conducted in the U.S. by Torres et al. [33] analyzed the salivary microbiome of 108 patients, 8 diagnosed with PDAC, 78 with other diseases and 22 healthy controls. Bacterial 16S rRNA V3-V4 region was amplified directly from salivary DNA extractions and subjected to sequencing. Despite the small cohort of PDAC patients in this study, a significantly higher ratio of *Leptotrichia* to *Porphyromonas* was reported in the saliva of PDAC patients compared to healthy subjects and patients with a variety of other diseases (including non-pancreatic cancer), and this ratio was suggested as a simple biomarker for PDAC [33]. Like Farrell et al., this study reported a lower proportion of Neisseria in PDAC patient saliva compared with the healthy and the other disease category, though this trend was not significant while the results for the other bacterial genera identified in the study by Farrell et al. were not confirmed. This discrepancy might be attributed to methodological differences (HOMIM microarray versus 16S rRNA V4 amplicon sequencing).

Another study conducted in the U.S. by Olson et al. [34] compared the microbiota composition of saliva in 40 PDAC patients, 39 patients with intraductal papillary mucinous neoplasms (IPMNs) and 58 healthy control subjects. Although PDAC cases did not differ in diversity measures from either controls or IPMN cases, they had higher mean relative proportions of Firmicutes and related taxa, while controls had higher mean relative proportions of Proteobacteria and related taxa. The mean relative proportion of taxa among IPMN patients was between PDAC patients and the controls [34]. The main limitations of this pilot study are the small sample size and a cross-sectional character of the study, as the oral samples were taken at only one time.

A study conducted in China by Lu et al. [35] investigated tongue coat microbiome composition in 30 PDAC patients with pancreatic cancer localized in the pancreatic head and 25 healthy subjects using 16S rRNA V3–V4 amplicon sequencing. Compared to controls, significantly increased tongue coat microbiome diversity in PDAC patients was found, with Fusobacteria as the most abundant phylum. Interestingly, *Haemophilus*, *Porphyromonas*, *Leptotrichia* and *Fusobacterium* could distinguish PDAC patients from healthy subjects in this study [35].

In a very recent case-control study, conducted in Iran by Vogtmann et al. [36], the oral microbiota composition of saliva was studied in 273 PDAC cases and 285 controls. Similarly to reports of Torres et al. and Olson et al., no association was observed for alpha diversity; however, there was evidence for an association between beta diversity and case status confirmed by multiple methods. There were also indications for associations between specific taxa and PDAC. Increasing relative levels of *Haemophilus* were associated with decreased odds of pancreatic cancer while the presence of Enterobacteriaceae, Lachnospiraceae G7, Bacteroidaceae and Staphylococcaceae were associated with increased odds of pancreatic cancer. This study has several limitations. Saliva samples were collected from pancreatic cancer cases at the time of diagnosis, not allowing to distinguish whether any microbial associations were related to pancreatic cancer etiology or the presence of disease. Second, controls were identified within patients who were also referred for endoscopic ultrasonography, thereby not representing healthy individuals who may have experienced microbial changes from underlying conditions. Third, despite the large sample size, this study was still underpowered for the taxa-specific analyses. Finally, information regarding oral health or tooth loss in this population was not obtained, and therefore the adjustment for potential confounding by these factors could not be addressed.

In a prospective study conducted on a large European cohort of 405 PDAC cases and 416 matched controls, Michaud et al. [37] measured antibodies to 25 oral bacteria in prediagnostic blood samples. A two-fold increase of cancer risk was observed in patients with high levels of IgG antibodies (>200 ng/µL) to the periodontal pathogen *Porphyromonas gingivalis ATCC 53978* compared with those with lower antibody titers, after adjusting for known risk factors, including smoking. These findings suggest that individuals who have high levels of antibodies to *P. gingivalis ATCC 53978* are at a two-fold higher risk of PDAC [37]. A large, nested case-control study conducted by Fan et al. [38] on a population-based cohort evaluated the microbiota profile in mouthwash samples of 361 PDAC patients and 371 matched healthy controls by 16S rRNA V3-V4 amplicon sequencing and associated *P. gingivalis* and *A. actinomycetemcomitans* with a higher risk of PDAC, while Fusobacteria and *Leptotrichia* were associated with a decreased tumor risk [38]. Unlike other studies mentioned above [32,33,34], this study was prospective, as saliva samples had been collected up to 10 years prior to cancer diagnosis, allowing the opportunity to determine the temporal relationship between oral microbiota and subsequent development of pancreatic cancer. Additionally, a large cohort included in the study provided sufficient statistical power to detect relevant associations between hypothesized explanatory factors and pancreatic cancer risk. Furthermore, the analysis was adjusted for known risk factors for PDAC like age, race, sex, smoking status, alcohol consumption, BMI and history of diabetes.

In summary, several studies report a potential link between oral microbiota and PDAC; however, no conclusive data on a specific bacterial species are yet available.

The inconsistency in results may partially be attributed to differences in methods and study designs. Geographic localization seems to play an important role in the composition of the microbiome of individuals [39,40,41,42]. Significant variations in microbiome composition in healthy individuals of different races and ethnicities and lifestyle-specific variations have been shown in several studies (reviewed in [39]). Four of the reviewed studies have been conducted in the U.S. and three in Europe, China and Iran, respectively, which might account for discrepancies observed. A small sample size might undermine the validity of a study and limit adjustments for certain potential confounders. Furthermore, different types of samples were analyzed, including saliva [32,33,34,36], tongue coating [35] and mouth washing [38]. Nevertheless, bacterial profiles have been found comparable in salivary and oral washing samples in a study that included 10 healthy individuals [43]. Oral microbiota composition and variety can be greatly affected by antibiotics. Three studies excluded participants using antibiotics from 2 to 12 weeks prior to sample collection [33,34,35], while other studies, to the best of our knowledge, did not address antibiotics usage in study participants. The DNA extraction method used seems to have a strong influence on the oral microbial diversity detected [44]. Targeted 16S rRNA amplicon sequencing is often a method of choice for oral microbiota profiling. Nevertheless, despite its advantages for analyzing samples with low biomass and high host genome contamination, it provides limited information at the species level. Additionally, the choice of primers and V1-V9 regions of the gene that will be amplified has been shown to influence the taxonomic profiling data in several studies [45,46,47]. Classification rate and accuracy might vary as different primers hit different proportions of sequences and the targeted regions are variably informative. All this might explain some confounding results reported by different studies.

Despite those limitations, oral *Porphyromonas gingivalis* and *Fusobacterium nucleatum* emerge as new risk factors for PDAC. However, the experimental evidence demonstrating *P. gingivalis*-induced PDAC progression is still missing. *P. gingivalis* plays a critical role in initiating inflammation via various virulence factors supported by the evidence that individuals with periodontal disease manifest elevated markers of systemic inflammation [48]. Interestingly, a recent investigation found higher loads of oral bacterial DNA in the cyst fluid of intraductal papillary mucinous neoplasms that could lead to the development of PDAC [49]. Additionally, *Fusobacterium* species were detected in pancreatic cancer tissue and *Fusobacterium* intratumoral status has been shown independently associated with a worse prognosis in pancreatic cancer patients, suggesting the potential to be used as a prognostic biomarker for pancreatic cancer [50].

Finally, *Neisseria elongata* and *Streptococcus mitis* decrease in abundance has been found to be significantly associated with PDAC in several studies [32,33,34].

To conclude, oral microbiota has the potential to be used as a non-invasive diagnostic and prognostic biomarker in PDAC and merits further exploration and confirmation, in prospective, multicenter, large cohort studies with a focus on the effects of geographical location in addition to known PDAC risk factors. Using a longitudinal, sampling approach in future studies might allow evaluating how oral microbiota composition patterns influence PDAC development and disease progression.

## 3. Gastrointestinal Microbiota and PDAC

### 3.1. Gastric Microbiota and PDAC-Related Helicobacter pylori

The long-held view of the stomach as a sterile organ has been changed after the discovery of *Helicobacter pylori* [51]. Due to its acidic conditions, the stomach has a particular composition of bacteria, different from bacteria in other gastrointestinal (GI) segments.

*H. pylori* is a gram-negative bacterium that colonizes the human stomach and infects nearly 50% of the world’s population. *H. pylori* yield various virulence factors that may alternate host intracellular signaling and influence the neoplastic transformation: *H. pylori* strains that express cytotoxin-associated gene A (Cag-A) are associated with gastric inflammation and ulceration and promote malignant transformation in gastric cancer [52,53]. Because of its direct influence on gastric carcinogenesis, *H. pylori* was the first bacterium to be considered carcinogenic and classified as a class 1 carcinogen by the International Agency for Research on Cancer (IARC) [54].

*H.pylori* infections have been associated with increased risk for PDAC in several case-control and prospective cohort studies [55,56,57,58,59]. However, some studies have found no relationship [60,61] and a possible role for *H. pylori* infection in pancreatic disease remains still controversial.

An antigenic peptide of *H. pylori* was identified in patients with autoimmune pancreatitis and PDAC [62]. A case-control study confirmed the presence of the *Helicobacter* genus in 75% of PDAC patients tested but not in pancreatic controls with benign disease [63] (Table 2). The proposed mechanisms of dissemination were hepatobiliary translocation or haematogenous seeding; however, mutually exclusive *Helicobacter* species have been identified in the same patients’ duodenal and PDAC tissue, making gut translocation of *Helicobacter* unlikely [63].

Several studies explored the possible indirect mechanisms by which *H. pylori* might engender PDAC, including inflammation, immune escape and exposure to elevated levels of nitrosamines [70,71]. In a study by Takayama and colleagues, infection of a human pancreatic cell line with *H. pylori* promoted upregulation of NF-*κ*B and pro-inflammatory cytokines, like IL-8, that are activating signaling pathways implicated in PDAC initiation and progression [72].

### 3.2. Gut Microbiota

The gastrointestinal tract is the largest microbial compartment in the body, and the gut microbiota contains over 10^14^ microorganisms, belonging mostly to the phyla Firmicutes and Bacteroidetes, and around 3 million genes, encoding enzymes that generate metabolites that can influence human physiology and pathophysiology [73,74].

Balanced gut microbial communities positively contribute to the host immune system and maintain immune homeostasis. Gut microbial dysbiosis is a change in the regular microbiome composition that can initiate chronic inflammation, epithelial barrier breaches and overgrowth of harmful bacteria, all shown to contribute to cancerogenesis [75].

The gut microbial dysbiosis has been proven linked to several GI inflammatory diseases, including coeliac disease, irritable bowel syndrome, inflammatory bowel disease, as well as colorectal cancer [23]. Apart from the direct effects of alterations of the microbiome gut, dysbiosis may cause long-distance effects in other organs such as the liver, breast, lung and pancreas [76].

Several studies have emphasized the difference in the gut microbiota composition between PDAC patients and healthy individuals (Table 3).

In a case-control study including 85 PDAC patients and 57 matched healthy controls by Ren et al. [77], a stool was collected to analyze microbial characteristics. Results show that gut microbial diversity was decreased in PDAC with a unique microbial profile, increased *Bacteriodetes* and decreased *Firmicutes* and *Proteobacteria* in PDAC cases compared to healthy controls suggesting its use for non-invasive PDAC diagnosis [77].

The analysis of the duodenal mucosal microbiota in a small case-control study including 14 patients with pancreatic head cancer and 14 healthy controls revealed that the microbiota of the duodenal mucosa in PDAC patients and healthy controls shared similar species, mostly Firmicutes and Proteobacteria phyla. However, duodenal samples of PDAC patients were characterized by enrichment with *Acinetobacter, Aquabacterium*, *Oceanobacillus and Rahnella*. In contrast, the duodenal microbiota of healthy controls were enriched with *Porphyromonas*, *Paenibaccilus*, *Enhydrobacter*, *Escherichia*, *Shigella* and *Pseudomonas* [78]. A very recent study compared bacterial and fungal (16S and 18S rRNA) profiles of secretin-stimulated duodenal fluid collections from 308 patients undergoing duodenal endoscopy including 134 normal pancreas controls, 98 patients with pancreatic cyst(s) and 74 patients with PDAC [79]. Patients with PDAC had significantly decreased duodenal microbial alpha diversity with an enrichment of *Bifidobacterium* genera compared to age-matched controls with normal pancreata and those with pancreatic cyst(s). Duodenal fluid microbiome profiles were not significantly different between controls and patients with pancreatic cyst(s). Additionally, it has been observed that duodenal fluid in PDAC patients with short-term survival was enriched with Fusobacteria and Rothia [79]. The authors also found higher levels of fungal DNA in patients with pancreatic cancer and higher levels of Ascomycota in duodenal fluid samples from patients with pancreatic cysts.

A study on 12 PDAC patients reported a higher abundance of Proteobacteria, Actinobacteria, Fusobacteria and Verrucomicrobia in the gut of PDAC patients [80].

In an effort to address the specific question of whether the pancreas has its own microbiome, Del Castillo et al. [66] (Table 2 and Table 3) performed 16S rRNA gene sequencing on 189 tissue samples (pancreatic duct, duodenum, pancreas), 57 swabs (bile duct, jejunum, stomach) and 12 stool samples. Bacterial DNA across different sites in the pancreas and duodenum overlapped and were highly subject-specific in both cancer and noncancer subjects. The relative abundance of *Fusobacterium* spp. was higher in cancer subjects compared to noncancer subjects, and the presence of genus *Lactobacillus* was significantly higher in noncancer subjects compared with cancer subjects. A higher prevalence of *Bifidobacterium* was detected in pancreatic/duodenal tissue samples from PDAC subjects compared with controls. The authors also detected a higher abundance or oral pathogens (*Porphyromonas*) in PDAC tissues compared to controls.

In conclusion, gut microbial diversity (alpha diversity) has been shown to be significantly decreased in PDAC patients compared to healthy subjects. Again, as previously mentioned, different sizes and designs of the studies, sampling methods and the choice of 16S rRNA V regions that were amplified might account for some disparity between study results.

## 4. Intratumoral Microbiota

The pancreas has been a long time considered germ-free. One of the first studies that confirmed the presence of specific bacterial DNA in the pancreatic tissue of PDAC patients dates back to 2006, and it reported the presence of *Helicobacter pylori* DNA in PDAC tissue [63].

Since then, the presence of diverse bacterial populations was confirmed in several studies in healthy pancreatic tissue, a cystic precursor that might lead to PDAC as well as in the intratumoral tissue itself (Table 2).

In patients undergoing pancreaticoduodenectomy, Rogers et al. [64] examined associations between microbiome data and preoperative biliary stent placement, neoadjuvant chemotherapy, postoperative pancreatic leak and death at 1 year. Fluids were collected from the bile duct, jejunum, pancreas and pancreatic cysts. Many bacterial taxa were detected in fluids obtained from the pancreatic ducts and the common bile duct, including *Prevotella, Haemophilus, Aggregatibacter* and *Fusobacterium*. The authors observed a preponderance of *Klebsiella* species (most commonly *K. pneumonia* and *K. oxytoca*) in PDAC patients and a decrease in beneficial and potentially anti-inflammatory strains *Faecalibacterium prausnitzii* and *Roseburia* spp. in fecal samples confirming that the gut microbiome is highly abnormal in the postoperative period. Additionally, they found that preoperative stent placement for biliary obstruction was associated with increased abundance of *Acinetobacter* and *Sphingobium* in pancreatic samples and decreased abundance of *Haemophilus* within the bile. Neoadjuvant therapy was associated with decreased *Bifidobacterium* in pancreatic fluid, increased *Bacteroides* and *Megasphaera* within the bile and increased *Clostridium* and *Enterococcus* within fecal samples. Finally, death at 1 year was associated with decreased *Klebsiella* within fecal samples [64].

Pancreatic cystic neoplasms (PCNs) include different types of cysts with various biological behavior. The most prevalent PCNs are intraductal papillary mucinous neoplasms (IPMNs) that can degenerate into full-blown PDAC. A study conducted on 27 PDAC, 21 non-IPMN cysts and 57 IPMN cysts has shown that intracystic bacterial 16S DNA copy numbers were significantly higher in IPMNs with high-grade dysplasia and IPMNs with cancer compared with non-IPMN PCNs. Even though the high interpersonal variation of intracystic microbiota composition has been detected, an enrichment of oral bacterial taxa, including *Fusobacterium nucleatum* and *Granulicatella adiacens,* has been found in cyst fluid from IPMNs with high-grade dysplasia [49].

Several other studies have shown an increased abundance of microorganisms in PDAC tissues, compared to non-neoplastic, healthy pancreatic tissues. Species belonging to the *Proteobacteria* and *Firmicutes* phyla accounted for most of the detected bacterial sequences in PDAC, similarly to the composition of the healthy gut microbiome [65,68,80].

Finally, Aykut et al. investigated the presence of mycobiota in pancreatic cancer, and they showed that human PDAC samples are markedly enriched for *Malassezia* spp. [69].

### Intratumoral Microbiota as a Prognostic Biomarker for PDAC

An interesting recent study has shown that intratumoral microbiome composition can be an indicator of PDAC patients’ survival [67]. By examining tumor DNA samples from PDAC patients with different survival outcomes by targeted 16S rRNA amplicon sequencing, Riquelme et al. [67] (Table 2) found that long-term survivors (i.e., five years or more of overall survival) have higher intratumoral microbiome diversity as compared to short-term survivors. They have identified an intratumoral microbiome signature (*Pseudoxanthomonas-Streptomyces-Saccharopolyspora-Bacillus clausii*) to be highly predictive of long-term survival. Both cohorts, long-term PDAC survivors (LTSs) and short-term PDAC survivors (STSs), were age-, gender-, tumor stage- and treatment-matched. Furthermore, by performing human-into-mice fecal microbiota transplantation from long-term survival donors, they were able to induce tumor shrinkage and activation of the immune system in tumor-bearing mice, confirming the ability of the gut microbiome to influence and modulate the PDAC tumor microbiome. They demonstrated that mice tumors had significantly higher numbers of CD8^+^ T cells, activated T cells (CD8^+^IFNg^+^ T cells) as well as a higher serum level of interferon-γ (IFN-γ) and interleukin-2 (IL-2) compared to mice that received fecal microbiota transplantation from short-term survivors. Furthermore, they found that CD8^+^ T cell depletion blocked this anti-tumoral effect suggesting this beneficial effect is mediated by CD8^+^ T cells [67].

The intrapancreatic abundance of oral *Fusobacterium* species was found to be relatively higher in PDAC patients than in noncancer controls in several studies, and this was independently associated with worse patient survival [50,66]. However, the analysis of the Mitsuhashi et al. [50] was not adjusted for treatment data which may skew the results.

In a study reported by Rogers et al. [64] (Table 2), death at 1 year in PDAC patients was associated with decreased *Klebsiella* within fecal samples.

Together, these data strongly suggest that the tumor microbiome diversity might be useful as a prognostic tool determining the survival of PDAC patients. Additionally, fecal microbiota transplantation results are opening a new therapeutic possibility to improve the survival of PDAC patients by manipulating their microbiome.

## 5. Microbiota Studies in Animal Models Are Paving the Way for Future Microbiota-Based Treatment Strategies for Pancreatic Cancer

Studies in genetically engineered mouse models of pancreatic neoplasia have revealed evidence for tumor-promoting effects of the gut microbiota on PDAC [80,81,82] (Table 4). Fluorescently labeled bacteria induced by oral gavage in germ-free mice have been shown to invade the pancreas, confirming the hypothesis of gut microbiota translocation to the pancreas [80].

The depletion of the intestinal bacterial microbiota in antibiotic-treated mice has been shown to reduce the development of PDAC in several studies [81,82]. Moreover, fecal microbiome transplantation in germ-free mice induced changes in immune cells within the PDAC tumor microenvironment [67,80].

Resistance to currently used chemotherapy regimens for PDAC remains the main obstacle for clinicians. Accumulating evidence from animal models suggests that microbiota might be involved in chemotherapy resistance. In a mouse model of colon cancer, Geller et al. [65] found that *Mycoplasma hyorhinis* (*M. hyorhinis*), belonging to Firmicutes phylum, can metabolize the chemotherapeutic drug gemcitabine (2′,2′-difluorodeoxycytidine) into its inactive form, 2′,2′-difluorodeoxyuridine. Metabolism was dependent on the expression of a long isoform of the bacterial enzyme cytidine deaminase (CDD_L_), seen primarily in Gammaproteobacteria. They further showed that gemcitabine resistance was induced by intratumoral Gammaproteobacteria that express CDD_L_ and could be abrogated by a co-treatment with the antibiotic ciprofloxacin. Since gemcitabine is the first-line treatment for PDAC, they further explored the microbiota of PDAC tissues and discovered that 76% of tested PDAC samples contained intratumoral Gammaproteobacteria class, suggesting these bacteria contribute to a gemcitabine resistance, prominent in PDAC patients. To confirm that bacteria derived from human PDAC can mediate gemcitabine resistance, they cultured bacteria from 15 fresh human PDAC tumors and found that 14/15 (93%) rendered the human colon carcinoma cell lines fully resistant to gemcitabine. These results indicate that PDACs contain bacteria that can potentially modulate tumor sensitivity to gemcitabine.

By repopulating germ-free Ptf1a^Cre^; LSL-Kras^G12D^ (KC) mice with feces from pancreatic cancer-bearing Ptf1a^Cre^; LSL-Kras^G12D^; Trp53^R172H^ (KPC) mice, Pushalkar et al. accelerated disease progression in KC mice [80]. Similarly, repopulating KC mice with *Bifidobacterium* *pseudolongum* accelerated cancer progression. *B. pseudolongum* could also be detected within the pancreata of treated KC mice, suggesting the bacterial translocation from the gut into the pancreas. They further showed that depletion of the gut microbiome with antibiotics reshaped tumor microenvironment by the diminution of myeloid-derived suppressor cell (MDSC) infiltration and reprogramming of tumor-associated macrophages toward a tumor-protective M1-like phenotype, accentuating Th1 polarization of CD4^+^ T cells and enhancing the cytotoxic phenotype of CD8^+^ T cells. Antibiotics up-regulated PD-1 expression on tumor-infiltrating CD4+ and CD8+ T cells which sensitize tumors to anti-PD-1 based immunotherapy. The authors suggest oral antibiotics in combination with checkpoint-directed immunotherapy as an attractive strategy for experimental therapeutics in PDAC patients.

Similarly, Sethi et al. [82] demonstrated in several mouse models that gut microbiome modulation may have an impact on PDAC tumor growth. First, a cocktail of broad-spectrum antibiotics was orally administered to Rag1 knock-out (mouse model lacking mature T and B cells), Kras^G12D/+^; Trp53^R172H/+^; Pdx1^cre^ (KPC) and Pten^fl/fl^ mice for 15 days. After two weeks, a pancreatic cell line derived from KPC mice was injected subcutaneously or intrasplenically (to induce liver metastasis). The absence of gut microbiota led to a significant decrease of pancreatic tumor burden in all models except Rag1 KO, which lack mature T and B cells, or mice treated with interleukin 17A (IL17a)-neutralizing antibody, thereby, suggesting that a direct cytotoxic effect of antibiotics is not responsible for mediating the anti-tumor phenomenon. Flow cytometry analyses demonstrated that gut microbiome depletion led to a significant increase in IFNγ-producing T cells with a corresponding decrease in IL17A and IL10-producing T cells [82].

Thomas et al. [81] showed an acceleration of the natural progression of PanIN to PDAC in the Kras^G12D^/PTEN^lox/+^ murine model of PDAC; there was a greater incidence of cancer formation, particularly poorly differentiated cancers, when a microbiota was present. They identified intestinal biota as an important mediator of pancreatic cancer progression since increased PDAC xenograft tumorigenicity was observed in microbiota-intact mice compared to microbiota-depleted mice [81].

Besides bacteria, a very recent study showed that fungi may also have a role in PDAC. Aykut et al. [69] (Table 2 andTable 4) found an increased abundance of fungi in tumors from both patients with PDAC and genetically engineered mouse models of pancreatic neoplasia relative to normal pancreatic tissue of healthy individuals and wild-type mice. To test how the pancreas gets infiltrated with fungi, the authors administered fluorescently tagged *Saccharomyces cerevisiae* to wild-type and PDAC mice via oral gavage and demonstrated that within 30 min labeled fungi could be found in the pancreas. The gut and pancreatic duct are directly linked by the sphincter of Oddi, which suggested to the researchers that translocation of the gut mycobiota could occur via this route. Sequencing analyses of fungal DNA from the gut and pancreatic tissues of mice revealed compositional differences between gut and tumor mycobiome. Overall, tumor tissue exhibited reduced taxonomic diversity but was enriched for *Parastagonospora*, *Saccharomyces*, *Septoriella* and *Malassezia* genera. Identical observations were made by sequencing tumor tissue and fecal samples from patients with PDAC, validating the mouse data. Once the mice underwent antifungal treatment with amphotericin B or fluconazole to kill off the fungal species in their pancreases, the authors repopulated them with specific fungus species to determine which species specifically caused cancer growth. They found that oncogenesis was accelerated in mice repopulated with *Malassezia* but not *Candida*, *Saccharomyces* or Aspergillus. Additionally, ablation of the mycobiome with the antifungal drugs protected against the PDAC progression and enhanced the effects of gemcitabine by 15 to 25 percent. The authors further found that fungi increase cancer risk by activating the complement cascade [69].

These studies in animal models provide substantial evidence that changes in gut microbiota induced by antibiotics influence PDAC progression and PDAC sensitivity to chemotherapy and immunotherapy, which could instruct the development of novel combinatorial antibiotic and immunotherapeutic strategies. In this respect, several interventional clinical trials on PDAC patients are currently ongoing testing the use of microbiota to optimize drug efficacy and/or reduce side effects (Table 5).

## 6. Conclusions and Perspectives

It is well established that the microbiome affects many vital functions in the human body. The alterations in the microbiome have been associated with a variety of diseases, including PDAC. After a reliable number of epidemiological studies indicating a relationship between the microbiome and pancreatic cancer was initially published, the mechanisms underlying this relationship are increasingly being revealed, but we are still far from understanding the whole picture. One of the main questions that remain to be answered: does microbiota play a causal role in the development of cancer, or does its presence reflect infections of already established tumors?

Recent discoveries described in this review challenge current physiological models of the gastrointestinal tract that assume that oral cavity, gut and solid tumors harbor mostly independent and segregated microbial communities. Identification of oral and gut microbiota members in the PDAC tissue, as well as the evidence from the animal models of microbiota translocation from the gut into the pancreas, allow presuming transmission of microbiota from the oral cavity, through the gastrointestinal tract into the solid tumors. Further studies would be needed to understand better this process and pathways of dissemination and their eventual influence on tumorigenesis. For example, the exploration of salivary, fecal and intratumoral microbiota in the same patient might be informative in this regard. Even though the initial focus in microbiome studies related to cancer was predominantly on bacteriome, the biggest constituent of the human microbiome, a very recent report suggests that mycobiota composition and changes are also involved in the pathogenesis of PDAC by promoting pancreatic inflammation. In light of this new data, it is of utmost interest to further explore various microbial communities and their relationships during PDAC tumorigenesis as well as the impact and the consequences of therapeutic targeting of one microbial community on the others. In addition to several above-mentioned limitations, targeted 16S rRNA amplicon sequencing analysis is not useful and applicable for the detection of the members from other kingdoms, like fungi and viruses, for example. Shotgun metagenomic sequencing, which sequences all the genomic DNA in a sample, allows detecting non-microbial reads, belonging to viruses, fungi and protists in addition to microbial taxa. Additionally, microbial genes present in the sample can be identified and profiled which gives additional information about microbiome functional potential. However, even though shotgun metagenome sequencing provides more information than 16S rRNA sequencing, it has several limitations including a relatively high cost and a more complex bioinformatics analysis that is necessary to obtain the results.

Certain microorganisms have been proven to induce resistance of PDAC to chemotherapy. This suggests that microbiome manipulation might have an excellent potential to overcome the current lack of an effective treatment for chemo-resistant PDAC. Additionally, microbiome manipulation has been shown to favorably affect the response of PDAC tumors to immunotherapy in animal models. Future research efforts are needed to better understand how microbiota impacts chemotherapy and immunotherapy in order to generate novel and personalized therapeutic approaches for PDAC patients. In this view, gut and intratumoral microbiome evaluation can be incorporated in our future clinical trials, and changes from baseline to post-therapeutic intervention should be monitored for a better comprehensiveness of chemo-immunotherapy susceptibility.

Finally, the shreds of evidence reviewed here give hope that in the near future, the analysis of the microbiome might lead to the validation of novel diagnostic and prognostic biomarkers that would improve the survival rates for patients with this insidious disease.

## Figures and Tables

**Table 1:** List of studies investigating the correlation between oral microbiota alterations and pancreatic ductal adenocarcinoma (PDAC). Pancreatic ductal adenocarcinoma (PDAC); number of subjects (N); healthy control (HC); chronic pancreatitis (CP); intraductal papillary mucinous neoplasm (IPMN); Human Oral Microbe Identification Microarray (HOMIM); the area under the parasitemia curve (AUC).

Ref.	Study Design	N. PDAC Patients	N. Controls	Detection Method	Microbiota Association	Main Findings/Authors’ Conclusion
Farrell et al., 2012 [32]	Case-control	38	38 HC 27 CP	Saliva, 16S rRNA amplicon hybridized on HOMIM array	*Granulicatella* adiacens increased, *Neisseria elongata* and *Streptococcus mitis* decreased abundance in PDAC cases	Combination of salivary *N. elongata* and *S. mitis* abundance distinguished cancer patients from healthy controls (AUC = 0.90) and proposed as a specific biomarker for PDAC. *G. adiacens* and *S. mitis* distinguished cancer patients from chronic pancreatitis (AUC = 0.68).
Torres et al., 2015 [33]	Case-control	8	22 HC	Saliva, 16S rRNA V3-V4 amplicon sequencing	*Leptotrichia* and *Porphyromonas* increased abundance *Neisseria* and *Aggregatibacter* decreased abundance in PDAC	Overall microbiota diversity of the groups was very similar. Salivary *Leptotrichia* to *Porphyromonas* ratio proposed as PDAC biomarker.
Olson et al., 2017 [34]	Case-control	40	58 HC 39 IPMN	Saliva, 16S rRNA V4–V5 amplicon sequencing	Increased abundance of Firmicutes and related taxa in PDAC versus higher levels of Proteobacteria and related taxa in healthy controls	No differences in overall saliva microbiota diversity (alpha diversity) between PDAC and IPMN patients.
Lu et al., 2019 [35]	Case-control	30	25 HC	Tongue swab, 16S rRNA V3–V4 amplicon sequencing	*Fusobacterium, Leptotrichia, Actinomyces, Corynebacterium, Rothia, Moraxella* and *Atopobium* preponderance in PDAC	*Haemophilus, Porphyromonas, Leptotrichia* and *Fusobacterium* could distinguish PDAC patients from healthy subjects (AUC = 0.802).
Vogtmann et al., 2020 [36]	Case-control	273	285 HC	Saliva, 16S rRNA V4 amplicon sequencing	Increased abundance of Enterobacteriaceae, Lachnospiraceae G7, Bacteroidaceae or Staphylococcaceae and decreased abundance of *Haemophilus* associated with PDAC	No differences in overall saliva microbiota diversity (alpha diversity) between PDAC and HC. Significant association between PDAC and microbial community composition (beta diversity).
Michaud et al., 2013 [37]	Prospectivenested case-control	405	410 HC	Plasma (blood) samples, ELISA	*Porphyromonas gingivalis ATCC 53978* increased IgG in PDAC	Individuals with high levels of antibodies to *P. gingivalis ATCC 53978* are at a 2-fold higher risk of developing PDAC.
Fan et al., 2018 [38]	Prospectivenested case-control study	361	371 HC	Mouthwash, 16S rRNA V3-V4 amplicon sequencing	*P. gingivalis* and *A. actinomycetemcomitans* increased abundance	*P. gingivalis* and *A. actinomycetemcomitans* were associated with a higher risk of PDAC, while Fusobacteria and *Leptotrichia* were associated with a decreased risk.

**Table 2:** Studies investigating PDAC intratumoral microbiota. Number of subjects (N); pancreatic ductal adenocarcinoma (PDAC); healthy control (HC); chronic pancreatitis (CP); pancreatic neuroendocrine tumor (NET); pancreatic cyst (PCy); intraductal papillary mucinous neoplasm (IPMN); long-term PDAC survivor (LTS); short-term PDAC survivor (STS).

Ref.	Study Design	N. PDAC Patients	N. Controls	Detection Method	Microbiota Association	Main Findings/Authors’ Conclusion
Nilsson et al., 2006 [63]	Case-control study	40	7 HC 5 CP 14 NET 10 other	Surgical specimen, DNA genus-specific PCR	*Helicobacter* DNA detected in the pancreas of 75% of patients with adenocarcinoma but not detected in any control	*Helicobacter* DNA, mostly *H. pylori* genus, commonly detected in pancreatic cancer.
Rogers et al., 2017 [64]	Cross-sectional study	50	/	Pancreatic fluid, bile or jejunal contents, 16S rRNA V4 amplicon sequencing	An enrichment of *Klebsiella* spp. and a decrease in *Faecalibacterium prausnitzii* and *Roseburia* spp. in fecal samples of PDAC patients	*Klebsiella pneumoniae* and *Klebsiella oxytosa* prepondernace in PDAC. Death at 1 year was associated with decreased *Klebsiella* within fecal samples of PDAC patients.
Mitsuhashi et al., 2015 [50]	Cross-sectional study	283	/	Surgical specimen PCR	*Fusobacterium* increased in PDAC	Significantly shorter survival observed in the *Fusobacterium* species-positive group.
Geller et al., 2017 [65]	Cross-sectional study	65	20	Surgical specimen 16S rRNA V6 amplicon sequencing	Increased Proteobacteria in PDAC	Increased presence of Gammaproteobacteria in PDAC tissue contributes to gemcitabine resistance.
Gaiser et al. 2018 [49]	Case-control study	27	57 IPMN PCy 21 non-IPMN PCy	Cyst fluid and plasma samples 16S rRNA qPCR and full-length gene sequencing	Firmicutes and/or Proteobacteria increased in PDAC	Higher overall microbial diversity in PDAC.
Del Castillo et al., 2019 [66]	Cross-sectional study	31	18 CPPCy 8 other	16S rRNA V3-V4 amplicon sequencing	*Porphyromonas*, *Capnocytophaga*, *Prevotella*, *Selenomonas* and *Fusobacterium spp*. higher abundance in PDAC patients	The authors observed that pancreatic and gut microbiota are highly subject-specific and differ between PDAC and noncancer subjects.
Riquelme et al., 2019 [67]	Case-control study	37 LTS	31 STS	Tumor cores 16S rRNA V4 amplicon sequencing	*Pseudoxanthomonas-* *Streptomyces-* *Saccharopolyspora-Bacillus clausii* highly predictive of long-term survivorship	Fecal microbiome transfer from LTS patients reduced tumor growth in mice compared to STS patients.
Nejman et al., 2020 [68]	Retrospective study	67	/	Tumor cores 16S rRNA V6 amplicon sequencing	*Fusobacterium nucleatum* enrichment in PDAC	The human tumor microbiome is composed of tumor type-specific intracellular bacteria present in both cancer and immune cells.
Aykut et al., 2019 [69]	Case-control study	13	18 fecal samples	18S ITS1 amplicon sequencing	*Parastagonospora, Saccharomyces, Septoriella* and *Malassezia* genera enriched in PDAC patients	Human PDAC samples markedly enriched for *Malassezia* spp.

**Table 3:** Studies investigating the association of gut and fecal microbiota dysbiosis and PDAC. Number of subjects (N); pancreatic ductal adenocarcinoma (PDAC); healthy control (HC); chronic pancreatitis (CP); pancreatic cyst (PCy).

Ref.	Study Design	N. PDAC Patients	N. Controls	Detection Method	Microbiota Association	Main Findings/Authors’ Conclusion
Ren et al., 2017 [77]	Case-control study	85	57	Fecal samples, 16S rRNA V3-V5 amplicon MiSeq sequencing	Bacteroidetes significantly increased, Firmicutes and Proteobacteria decreased in PDAC compared to HC	Gut microbial diversity (alpha diversity) is significantly decreased in PDAC patients. Microbial alterations in PDAC present an increase of potentially pathogenic bacteria and a decrease of probiotics and butyrate-producing bacteria.
Mei et al., 2018 [78]	Case-control study	14	14	Duodenal mucosa 16S rRNA V3-V4 amplicon sequencing	Duodenal microbiota of PDAC patients enriched in *Acinetobacter, Aquabacterium, Oceanobacillus, Rahnella,* *Massilia,* *Delftia, Deinococcus* and *Sphingobium*	The results showed species in PDAC patients and HC belong mainly to the phyla Firmicutes and Proteobacteria.
Kohi et al., 2020 [79]	Case-control study	74	134 HC 98 PCy	16S V3-V4 and 18S ITS1 rRNA amplicon sequencing	Duodenal microbiota of PDAC patients enriched in *Escherichia-Shigella, Enterococcus, Clostridium* and *Bifidobacterium* and *Ascomycota* compared to HC	Patients with PDAC had significantly decreased duodenal microbial alpha diversity with an enrichment of *Bifidobacterium* compared to controls. An enrichment of duodenal fluid *Fusobacteria* and *Rothia* was detected in PDAC patients with short survival.
Pushalkar et al., 2018 [80]	Retrospective study	12	Not reported	Fecal samples and PDAC surgical specimen 16S rRNA V3-V4 amplicon sequencing	*Proteobacteria,* *Actinobacteria, Fusobacteria* and *Verrucomicrobia* higher abundance in the gut of PDAC patients	Gut microbiome indicated significant differences in bacterial abundances between Stage I/II and Stage IV PDAC patients.
Del Castillo et al., 2019 [66]	Cross-sectional study	31	18 CP PCy 8 other	Duodenal, PDAC and PCy samples 16S rRNA V3–V4 amplicon sequencing	*Porphyromonas,* *Capnocytophaga, Prevotella,* *Selenomonas,* *Fusobacterium* spp. higher abundance in PDAC patients	The authors observed that pancreatic and gut microbiota are highly subject-specific and differ between PDAC and noncancer subjects.

**Table 4:** Studies on animal models; KC mice: p48^Cre^; LSL-Kras^G12D^. KPC mice: Pdx1^Cre^; LSL-Kras^G12D^; p53R^172H^; Rag1. KO: C57BL/6J mice carrying a Rag1^tm1Mom^. WT: wild-type.

Ref.	Experimental Model	Microbiota Association	Main Findings/Authors’ Conclusion
Geller et al., 2017 [65]	Mouse model of colon cancer	Increased Gammaproteobacteria in PDAC	Gammaproteobacteria possessing a long isoform of cytidine deaminase can metabolize gemcitabine.
Pushalkar et al., 2018 [80]	KC and KPC mice	Bacteroidetes and Firmicutes and select Actinobacteria- and Deferribacteres-associated genera were more prevalent in the early-KPC and advanced-KPC cohorts compared with WT	Gut microbiota migrates into the pancreas in mice models. Germ-free mice are protected against PDAC progression. Modulation of gut microbiota influences PDAC tumor progression and has a potential to augment PDAC responsiveness to immune therapy.
Thomas et al., 2018 [81]	Kras^G12D^/PTEN^lox/+^ mice	*Acinetobacter, Enterobacter, Pseudomonas,* *Delftia, Enterococcus, Streptococcus,* *Corynebacterium, Propionibacterium,* *Klebsiella, Sphingomonas* and *Staphylococcus. Klebsiella* as disproportionally overrepresented in PDAC versus HC	Intestinal microbiota exerts long-distance modulation and enhances PDAC carcinogenesis in transgenic and xenograft mouse models of PDAC.
Sethi et al., 2018 [82]	Rag1 KO, KPC, Pten^fl/fl^ mice	Antibiotics induced a significant decrease in α-diversity, decrease in the relative abundance of Bacteroidetes and Firmicutes in the stool of KPC-bearing mice as well as reversed Bacteroidales: Clostridiales abundance ratio and colonization of the gut by likely antibiotic-resistant Proteobacteria and Tenericutes	Gut microbiome depletion significantly reduced tumor burden in all the models tested, except for Rag1-knockout mice, which lack mature T and B cells.
Aykut et al., 2019 [69]	KC, KPC and WT mice	*Malassezia*, at about 20% abundance *Malassezia* spp., most prevalent genus in pancreata of KC mice and exerts a tumor-promoting effect.	Ablation of the mycobiome with the antifungal drugs in mice protected against the PDAC progression and enhanced the effects of gemcitabine by 15 to 25 percent.

**Table 5:** Ongoing and completed interventional clinical trials testing the use of microbiota in combination with anticancer drugs in order to optimize drug efficacy or reduce side effects (source: https://clinicaltrials.gov, accessed on 24 February 2021).

NCT Number	Study Design	Intervention	Condition	Title	Number of Patients	Status
NCT04600154	Interventional	Drug: MS-20 Other: Placebo	Pancreatic Cancer	MS-20 on Gut Microbiota and Risk/Severity of Cachexia in Pancreatic Cancer Patients	40	Recruiting
NCT04447443	Interventional	Dietary Supplement: Prebiotic Fiber Supplement + loperamide hydrochloride capsule Dietary Supplement: Maltodextrin + loperamide hydrochloride capsule	Gastrointestinal Tumors	Impact of Dietary Fiber as Prebiotics on Chemotherapy-related Diarrhea in Patients With Gastrointestinal Tumors	120	Recruiting
NCT04363983	Interventional	Biological: Blood sampling Procedure: Liver biopsy Biological: Stool collect	Gastrointestinal Neoplasms	Interaction Between Host, Microenvironment and Immunity on Gastrointestinal Neoplasms (HoMING)	6300	Not yet recruiting
NCT04193904	Interventional Phase I	Drug: MRx0518 Radiation: Hypofractionated preoperative radiation	Pancreatic Cancer	A Study of Live Biotherapeutic Product MRx0518 With Hypofractionated Radiation Therapy in Resectable Pancreatic Cancer	15	Recruiting
NCT03891979	Interventional Phase IV	Drug: Pembrolizumab Drug: Ciprofloxacin 500 mg PO BID days 1–29 Drug: Metronidazole 500 mg PO TID days 1–29	Pancreatic Cancer	Gut Microbiome Modulation to Enable Efficacy of Checkpoint-based Immunotherapy in Pancreatic Adenocarcinoma	0	Withdrawn (suspended)
NCT03331562	Interventional Phase II	Drug: Pembrolizumab Drug: Paricalcitol Drug: Placebo	Pancreatic Cancer	A SU2C Catalyst^®^ Trial of a PD1 Inhibitor with or without a Vitamin D Analog for the Maintenance of Pancreatic Cancer	24	Completed

## Data Availability

No new data were created or analyzed in this study. Data sharing is not applicable to this article.

## References

[1] Siegel R.L., Miller K.D., Fuchs H.E., Jemal A. (2021). Cancer Statistics, 2021. CA. Cancer J. Clin..

[2] Midha S., Chawla S., Garg P.K. (2016). Modifiable and non-modifiable risk factors for pancreatic cancer: A review. Cancer Lett..

[3] Silverman D.T., Dunn J.A., Hoover R.N., Schiffiman M., Lillemoe K.D., Schoenberg J.B., Brown L.M., Greenberg R.S., Hayes R.B., Swanson G.M. (1994). Cigarette Smoking and Pancreas Cancer: A Case—Control Study Based on Direct Interviews. JNCI J. Natl. Cancer Inst..

[4] Bosetti C., Lucenteforte E., Silverman D.T., Petersen G., Bracci P.M., Ji B.T., Negri E., Li D., Risch H.A., Olson S.H. (2012). Cigarette smoking and pancreatic cancer: An analysis from the International Pancreatic Cancer Case-Control Consortium (Panc4). Ann. Oncol. Off. J. Eur. Soc. Med. Oncol..

[5] Iodice S., Gandini S., Maisonneuve P., Lowenfels A.B. (2008). Tobacco and the risk of pancreatic cancer: A review and meta-analysis. Langenbeck’s Arch. Surg..

[6] Arslan A.A., Helzlsouer K.J., Kooperberg C., Shu X.-O., Steplowski E., Bueno-de-Mesquita H.B., Fuchs C.S., Gross M.D., Jacobs E.J., LaCroix A.Z. (2010). Anthropometric Measures, Body Mass Index, and Pancreatic Cancer: A Pooled Analysis From the Pancreatic Cancer Cohort Consortium (PanScan). Arch. Intern. Med..

[7] Wang Y.-T., Gou Y.-W., Jin W.-W., Xiao M., Fang H.-Y. (2016). Association between alcohol intake and the risk of pancreatic cancer: A dose–response meta-analysis of cohort studies. BMC Cancer.

[8] Huxley R., Ansary-Moghaddam A., Berrington de González A., Barzi F., Woodward M. (2005). Type-II diabetes and pancreatic cancer: A meta-analysis of 36 studies. Br. J. Cancer.

[9] Bosetti C., Rosato V., Li D., Silverman D., Petersen G.M., Bracci P.M., Neale R.E., Muscat J., Anderson K., Gallinger S. (2014). Diabetes, antidiabetic medications, and pancreatic cancer risk: An analysis from the International Pancreatic Cancer Case-Control Consortium. Ann. Oncol..

[10] Stolzenberg-Solomon R.Z., Graubard B.I., Chari S., Limburg P., Taylor P.R., Virtamo J., Albanes D. (2005). Insulin, Glucose, Insulin Resistance, and Pancreatic Cancer in Male Smokers. JAMA.

[11] Ben Q., Cai Q., Li Z., Yuan Y., Ning X., Deng S., Wang K. (2011). The relationship between new-onset diabetes mellitus and pancreatic cancer risk: A case–control study. Eur. J. Cancer.

[12] Jansen R.J., Tan X.-L., Petersen G.M. (2015). Focus: A Multifaceted Battle Against Cancer: Gene-by-Environment Interactions in Pancreatic Cancer: Implications for Prevention. Yale J. Biol. Med..

[13] Wolpin B.M., Chan A.T., Hartge P., Chanock S.J., Kraft P., Hunter D.J., Giovannucci E.L., Fuchs C.S. (2009). ABO blood group and the risk of pancreatic cancer. J. Natl. Cancer Inst..

[14] Amundadottir L., Kraft P., Stolzenberg-Solomon R.Z., Fuchs C.S., Petersen G.M., Arslan A.A., Bueno-de-Mesquita H.B., Gross M., Helzlsouer K., Jacobs E.J. (2009). Genome-wide association study identifies variants in the ABO locus associated with susceptibility to pancreatic cancer. Nat. Genet..

[15] Solomon S., Das S., Brand R., Whitcomb D.C. (2012). Inherited Pancreatic Cancer Syndromes. Cancer J..

[16] Magnúsdóttir S., Heinken A., Kutt L., Ravcheev D.A., Bauer E., Noronha A., Greenhalgh K., Jäger C., Baginska J., Wilmes P. (2017). Generation of genome-scale metabolic reconstructions for 773 members of the human gut microbiota. Nat. Biotechnol..

[17] Ursell L.K., Metcalf J.L., Parfrey L.W., Knight R. (2012). Defining the human microbiome. Nutr. Rev..

[18] Sender R., Fuchs S., Milo R. (2016). Are we really vastly outnumbered? Revisiting the ratio of bacterial to host cells in humans. Cell.

[19] Lukens J.R., Gurung P., Vogel P., Johnson G.R., Carter R.A., McGoldrick D.J., Bandi S.R., Calabrese C.R., Walle L. Vande, Lamkanfi M. (2014). Dietary modulation of the microbiome affects autoinflammatory disease. Nature.

[20] David L.A., Maurice C.F., Carmody R.N., Gootenberg D.B., Button J.E., Wolfe B.E., Ling A.V., Devlin A.S., Varma Y., Fischbach M.A. (2014). Diet rapidly and reproducibly alters the human gut microbiome. Nature.

[21] Desai M.S., Seekatz A.M., Koropatkin N.M., Kamada N., Hickey C.A., Wolter M., Pudlo N.A., Kitamoto S., Terrapon N., Muller A. (2016). A dietary fiber-deprived gut microbiota degrades the colonic mucus barrier and enhances pathogen susceptibility. Cell.

[22] Akshintala V.S., Talukdar R., Singh V.K., Goggins M. (2019). The Gut Microbiome in Pancreatic Disease. Clin. Gastroenterol. Hepatol..

[23] Carding S., Verbeke K., Vipond D.T., Corfe B.M., Owen L.J. (2015). Dysbiosis of the gut microbiota in disease. Microb. Ecol. Health Dis..

[24] Sambo F., Finotello F., Lavezzo E., Baruzzo G., Masi G., Peta E., Falda M., Toppo S., Barzon L., Di Camillo B. (2018). Optimizing PCR primers targeting the bacterial 16S ribosomal RNA gene. BMC Bioinformatics.

[25] Aas J.A., Paster B.J., Stokes L.N., Olsen I., Dewhirst F.E. (2005). Defining the normal bacterial flora of the oral cavity. J. Clin. Microbiol..

[26] Genco R.J. (1996). Consensus report, periodontal disease; pathogenesis and microbial factors. Ann. Periodontol..

[27] Ahn J., Segers S., Hayes R.B. (2012). Periodontal disease, Porphyromonas gingivalis serum antibody levels and orodigestive cancer mortality. Carcinogenesis.

[28] Michaud D.S., Joshipura K., Giovannucci E., Fuchs C.S. (2007). A prospective study of periodontal disease and pancreatic cancer in US male health professionals. J. Natl. Cancer Inst..

[29] Ansai T., Takata Y., Yoshida A., Soh I., Awano S., Hamasaki T., Sogame A., Shimada N. (2013). Association between tooth loss and orodigestive cancer mortality in an 80-year-old community-dwelling Japanese population: A 12-year prospective study. BMC Public Health.

[30] Stolzenberg-Solomon R.Z., Dodd K.W., Blaser M.J., Virtamo J., Taylor P.R., Albanes D. (2003). Tooth loss, pancreatic cancer, and Helicobacter pylori. Am. J. Clin. Nutr..

[31] Hujoel P.P., Drangsholt M., Spiekerman C., Weiss N.S. (2003). An exploration of the periodontitis–cancer association. Ann. Epidemiol..

[32] Farrell J.J., Zhang L., Zhou H., Chia D., Elashoff D., Akin D., Paster B.J., Joshipura K., Wong D.T.W. (2012). Variations of oral microbiota are associated with pancreatic diseases including pancreatic cancer. Gut.

[33] Torres P.J., Fletcher E.M., Gibbons S.M., Bouvet M., Doran K.S., Kelley S.T. (2015). Characterization of the salivary microbiome in patients with pancreatic cancer. PeerJ.

[34] Olson S.H., Satagopan J., Xu Y., Ling L., Leong S., Orlow I., Saldia A., Li P., Nunes P., Madonia V. (2017). The oral microbiota in patients with pancreatic cancer, patients with IPMNs, and controls: A pilot study. Cancer Causes Control.

[35] Lu H., Ren Z., Li A., Li J., Xu S., Zhang H., Jiang J., Yang J., Luo Q., Zhou K. (2019). Tongue coating microbiome data distinguish patients with pancreatic head cancer from healthy controls. J. Oral Microbiol..

[36] Vogtmann E., Han Y., Caporaso J.G., Bokulich N., Mohamadkhani A., Moayyedkazemi A., Hua X., Kamangar F., Wan Y., Suman S. (2020). Oral microbial community composition is associated with pancreatic cancer: A case-control study in Iran. Cancer Med..

[37] Michaud D.S., Izard J., Wilhelm-Benartzi C.S., You D.-H., Grote V.A., Tjønneland A., Dahm C.C., Overvad K., Jenab M., Fedirko V. (2013). Plasma antibodies to oral bacteria and risk of pancreatic cancer in a large European prospective cohort study. Gut.

[38] Fan X., Alekseyenko A.V., Wu J., Peters B.A., Jacobs E.J., Gapstur S.M., Purdue M.P., Abnet C.C., Stolzenberg-Solomon R., Miller G. (2018). Human oral microbiome and prospective risk for pancreatic cancer: A population-based nested case-control study. Gut.

[39] Gupta V.K., Paul S., Dutta C. (2017). Geography, ethnicity or subsistence-specific variations in human microbiome composition and diversity. Front. Microbiol..

[40] Renson A., Jones H.E., Beghini F., Segata N., Zolnik C.P., Usyk M., Moody T.U., Thorpe L., Burk R., Waldron L. (2019). Sociodemographic variation in the oral microbiome. Ann. Epidemiol..

[41] Blekhman R., Goodrich J.K., Huang K., Sun Q., Bukowski R., Bell J.T., Spector T.D., Keinan A., Ley R.E., Gevers D. (2015). Host genetic variation impacts microbiome composition across human body sites. Genome Biol..

[42] Mason M.R., Nagaraja H.N., Camerlengo T., Joshi V., Kumar P.S. (2013). Deep sequencing identifies ethnicity-specific bacterial signatures in the oral microbiome. PLoS ONE.

[43] Fan X., Peters B.A., Min D., Ahn J., Hayes R.B. (2018). Comparison of the oral microbiome in mouthwash and whole saliva samples. PLoS ONE.

[44] Teng F., Darveekaran Nair S.S., Zhu P., Li S., Huang S., Li X., Xu J., Yang F. (2018). Impact of DNA extraction method and targeted 16S-rRNA hypervariable region on oral microbiota profiling. Sci. Rep..

[45] Tremblay J., Singh K., Fern A., Kirton E.S., He S., Woyke T., Lee J., Chen F., Dangl J.L., Tringe S.G. (2015). Primer and platform effects on 16S rRNA tag sequencing. Front. Microbiol..

[46] Walker A.W., Martin J.C., Scott P., Parkhill J., Flint H.J., Scott K.P. (2015). 16S rRNA gene-based profiling of the human infant gut microbiota is strongly influenced by sample processing and PCR primer choice. Microbiome.

[47] Gihring T.M., Green S.J., Schadt C.W. (2012). Massively parallel rRNA gene sequencing exacerbates the potential for biased community diversity comparisons due to variable library sizes. Environ. Microbiol..

[48] Li X., Kolltveit K.M., Tronstad L., Olsen I. (2000). Systemic diseases caused by oral infection. Clin. Microbiol. Rev..

[49] Gaiser R.A., Halimi A., Alkharaan H., Lu L., Davanian H., Healy K., Hugerth L.W., Ateeb Z., Valente R., Fernández Moro C. (2019). Enrichment of oral microbiota in early cystic precursors to invasive pancreatic cancer. Gut.

[50] Mitsuhashi K., Nosho K., Sukawa Y., Matsunaga Y., Ito M., Kurihara H., Kanno S., Igarashi H., Naito T., Adachi Y. (2015). Association of Fusobacterium species in pancreatic cancer tissues with molecular features and prognosis. Oncotarget.

[51] Marshall B., Warren J.R. (1984). Unidentified Curved Bacilli In The Stomach Of Patients With Gastritis And Peptic Ulceration. Lancet.

[52] Kalaf E.A., Al-Khafaji Z.M., Yassen N.Y., Al-Abbudi F.A., Sadwen S.N. (2013). Study of the cytoxin-associated gene a (CagA gene) in Helicobacter pylori using gastric biopsies of Iraqi patients. Saudi J. Gastroenterol. Off. J. Saudi Gastroenterol. Assoc..

[53] Chen S., Duan G., Zhang R., Fan Q. (2014). Helicobacter pylori cytotoxin-associated gene A protein upregulates α-enolase expression via Src/MEK/ERK pathway: Implication for progression of gastric cancer. Int. J. Oncol..

[54] (1994). Schistosomes, liver flukes and Helicobacter pylori. IARC Monographs on the Evaluation of Carcinogenic Risks to Humans.

[55] Raderer M., Wrba F., Kornek G., Maca T., Koller D.Y., Weinlaender G., Hejna M., Scheithauer W. (1998). Association between Helicobacter pylori infection and pancreatic cancer. Oncology.

[56] Stolzenberg-Solomon R.Z., Blaser M.J., Limburg P.J., Perez-Perez G., Taylor P.R., Virtamo J., Albanes D. (2001). Helicobacter pylori seropositivity as a risk factor for pancreatic cancer. J. Natl. Cancer Inst..

[57] Lindkvist B., Johansen D., Borgström A., Manjer J. (2008). A prospective study of Helicobacter pylori in relation to the risk for pancreatic cancer. BMC Cancer.

[58] Bao Y., Spiegelman D., Li R., Giovannucci E., Fuchs C.S., Michaud D.S. (2010). History of peptic ulcer disease and pancreatic cancer risk in men. Gastroenterology.

[59] Risch H.A. (2003). Etiology of pancreatic cancer, with a hypothesis concerning the role of N-nitroso compounds and excess gastric acidity. J. Natl. Cancer Inst..

[60] de Martel C., Llosa A.E., Friedmana G.D., Vogelman J.H., Orentreich N., Stolzenberg-Solomon R.Z., Parsonnet J. (2008). Helicobacter pylori infection and development of pancreatic cancer. Cancer Epidemiol. Prev. Biomarkers.

[61] Gawin A., Wex T., Ławniczak M., Malfertheiner P., Starzyńska T. (2012). Helicobacter pylori infection in pancreatic cancer. Pol. Merkur. Lek. organ Pol. Tow. Lek..

[62] Frulloni L., Lunardi C., Simone R., Dolcino M., Scattolini C., Falconi M., Benini L., Vantini I., Corrocher R., Puccetti A. (2009). Identification of a novel antibody associated with autoimmune pancreatitis. N. Engl. J. Med..

[63] Nilsson H.-O., Stenram U., Ihse I., Wadstrom T. (2006). Helicobacter species ribosomal DNA in the pancreas, stomach and duodenum of pancreatic cancer patients. World J. Gastroenterol..

[64] Rogers M.B., Aveson V., Firek B., Yeh A., Brooks B., Brower-Sinning R., Steve J., Banfield J.F., Zureikat A., Hogg M. (2017). Disturbances of the Perioperative Microbiome Across Multiple Body Sites in Patients Undergoing Pancreaticoduodenectomy. Pancreas.

[65] Geller L.T., Barzily-Rokni M., Danino T., Jonas O.H., Shental N., Nejman D., Gavert N., Zwang Y., Cooper Z.A., Shee K. (2017). Potential role of intratumor bacteria in mediating tumor resistance to the chemotherapeutic drug gemcitabine. Science.

[66] del Castillo E., Meier R., Chung M., Koestler D.C., Chen T., Paster B.J., Charpentier K.P., Kelsey K.T., Izard J., Michaud D.S. (2019). The Microbiomes of Pancreatic and Duodenum Tissue Overlap and Are Highly Subject Specific but Differ between Pancreatic Cancer and Noncancer Subjects. Cancer Epidemiol. Biomarkers Prev..

[67] Riquelme E., Zhang Y., Zhang L., Jenq R., Wargo J., Mcallister F. (2019). Tumor Microbiome Diversity and Composition Article Tumor Microbiome Diversity and Composition Influence Pancreatic Cancer Outcomes. Cell.

[68] Nejman D., Livyatan I., Fuks G., Gavert N., Zwang Y., Geller L.T., Rotter-Maskowitz A., Weiser R., Mallel G., Gigi E. (2020). The human tumor microbiome is composed of tumor type–specific intracellular bacteria. Science.

[69] Aykut B., Pushalkar S., Chen R., Li Q., Abengozar R., Kim J.I., Shadaloey S.A., Wu D., Preiss P., Verma N. (2019). The fungal mycobiome promotes pancreatic oncogenesis via activation of MBL. Nature.

[70] Risch H.A. (2012). Pancreatic cancer: Helicobacter pylori colonization, N-Nitrosamine exposures, and ABO blood group. Mol. Carcinog..

[71] Jesenofsky R., Isaksson B., Möhrcke C., Bertsch C., Bulajic M., Schneider-Brachert W., Klöppel G., Lowenfels A.B., Maisonneuve P., Löhr J.-M. (2010). Helicobacter pylori in autoimmune pancreatitis and pancreatic carcinoma. Pancreatology.

[72] Takayama S., Takahashi H., Matsuo Y., Okada Y., Manabe T. (2008). Effects of Helicobacter pylori infection on human pancreatic cancer cell line. Hepatogastroenterology.

[73] Plottel C.S., Blaser M.J. (2011). Microbiome and Malignancy. Cell Host Microbe.

[74] Cho I., Blaser M.J. (2012). The human microbiome: At the interface of health and disease. Nat. Rev. Genet..

[75] Schwabe R.F., Jobin C. (2013). The microbiome and cancer. Nat. Rev. Cancer.

[76] Gopalakrishnan V., Helmink B.A., Spencer C.N., Reuben A., Wargo J.A. (2018). The Influence of the Gut Microbiome on Cancer, Immunity, and Cancer Immunotherapy. Cancer Cell.

[77] Ren Z., Jiang J., Xie H., Li A., Lu H., Xu S., Zhou L., Zhang H., Cui G., Chen X. (2017). Gut microbial profile analysis by MiSeq sequencing of pancreatic carcinoma patients in China. Oncotarget.

[78] Mei Q.-X., Huang C.-L., Luo S.-Z., Zhang X.-M., Zeng Y., Lu Y.-Y. (2018). Characterization of the duodenal bacterial microbiota in patients with pancreatic head cancer vs. healthy controls. Pancreatology.

[79] Kohi S., Macgregor-Das A., Dbouk M., Yoshida T., Chuidian M., Abe T., Borges M., Lennon A.M., Shin E.J., Canto M.I. (2020). Alterations In The Duodenal Fluid Microbiome Of Patients With Pancreatic Cancer. Clin. Gastroenterol. Hepatol..

[80] Pushalkar S., Hundeyin M., Daley D., Zambirinis C.P., Kurz E., Mishra A., Mohan N., Aykut B., Usyk M., Torres L.E. (2018). The pancreatic cancer microbiome promotes oncogenesis by induction of innate and adaptive immune suppression. Cancer Discov..

[81] Thomas R.M., Gharaibeh R.Z., Gauthier J., Beveridge M., Pope J.L., Guijarro M.V., Yu Q., He Z., Ohland C., Newsome R. (2018). Intestinal microbiota enhances pancreatic carcinogenesis in preclinical models. Carcinogenesis.

[82] Sethi V., Kurtom S., Tarique M., Lavania S., Malchiodi Z., Hellmund L., Zhang L., Sharma U., Giri B., Garg B. (2018). Gut Microbiota Promotes Tumor Growth in Mice by Modulating Immune Response. Gastroenterology.

